# Opening access to the black box: The need for reporting on the global phosphorus supply chain

**DOI:** 10.1007/s13280-019-01240-8

**Published:** 2019-09-04

**Authors:** Claudiu-Eduard Nedelciu, Kristín Vala Ragnarsdóttir, Ingrid Stjernquist, Marie Katharine Schellens

**Affiliations:** 1 Department of Physical Geography, Stockholm University, 106 91 Stockholm, Sweden; 2 Institute of Earth Sciences, University of Iceland, Askja, Sturlugata 7, 101 Reykjavík, Iceland

**Keywords:** Global governance, Open access data, Phosphorus, Supply chain, Systems analysis

## Abstract

Phosphorus (P) is an essential macronutrient in agriculture; however, lack of reporting makes its supply chain a black box. By using literature synthesis on the P challenge, we identify four areas where the reporting process is problematic: P reserves and resources; P losses along the supply chain; P externalities; and access to data. We find that in these areas, the reporting system is inconsistent, inaccurate, incomplete, fragmented and non-transparent. We use systems analysis to discuss implications of reporting on the sustainability of the P supply chain. We find that reporting is essential for the achievement of global P governance and the human right to adequate food. It can also inform decision makers and other impacted stakeholders on policies on agriculture, food security, pollution and international conflict. An improved P reporting process also allows a better evaluation of global sustainability commitments such as the United Nations Sustainable Development Goals.

## Introduction

Phosphorus (P) is an essential macronutrient needed for food production and human life, yet it has no substitute (Cordell et al. 2009). Because P a main limiting factor to plant growth, access to industrially produced inorganic P fertilizers has been indispensable for the Green Revolution and the contemporary large-scale, high-productivity agriculture (Cordell and White 2014). Estimates from the UN point towards over 9 billion people by 2050 and a correlated food demand increase of 60% (FAO 2013) for the same period, which would in turn trigger an increase in global P demand.

More than 85% of the P in agriculture comes from processing mined phosphate rock (PR) (Cordell et al. 2009). Phosphate rock is a finite natural resource globally, distributed in a limited number of countries. According to the latest data of the United States Geological Survey (USGS), most PR reserves are found in Morocco and Western Sahara (71.5%), China (4.6%) and Algeria (3%) (USGS 2019). At present, production is led by China (52%), Morocco and Western Sahara (12%), USA (10%) and Russia (5%) (USGS 2019).

In 2007–2008, an 800% increase in P fertilizer prices sent a shockwave to the world market and food price soared (Cordell and White 2011). The event led to the emergence of a “phosphorus challenge” (ESPP 2013) and the concept of “peak phosphorus” (e.g. Ragnarsdottir et al. 2011), which called for action in sustainably managing a scarce resource. A controversial revision of world PR reserves by USGS from 16 GT in 2010 to 65 GT in 2011 led to a more moderate peak phosphorus discourse, but raised questions concerning the methodology behind PR resource reporting (Edixhoven et al. 2014). Subsequently, accessibility to affordable P became more visible on policy agendas. In the European Union (EU), P was included in the list of Critical Raw Materials in 2014 (European Commission 2014). The most recent report of the Food and Agriculture Organisation (FAO) with regard to fertilizer trends and outlook for 2020 (FAO 2017) also shows that a number of regions have a negative phosphate balance (reported as P_2_O_5_): Oceania, Central and Western Europe, South Asia and Latin America, and the Caribbean. The gap between supply and total demand is expected to further widen by 2020.

Other aspects of the phosphorus challenge were brought into discussion following the 2007–2008 price soaring event. It was acknowledged that the phosphorus supply chain demonstrates an unsustainable and linear use, with large fractions of waste at each stage, from mine to fork (Scholz and Wellmer 2015). P is also one of the main causes for eutrophication of water bodies across the world. Fertilizers entering the ocean are responsible for the creation of 400 coastal dead zones, totalling more than 245 000 km^2^ (UN 2019). Thus currently, P is both angel and demon: it is vital for agricultural productivity, yet it is one of the most widespread water pollutants, causing ecosystem devastation.

In the literature, academics have called for policy leadership and global governance for P, with a preferably UN-related institution to overlook reporting (Rosemarin and Ekane 2016). They argue that the reporting process could benefit from more harmonization of terminologies and methodologies, as well as become more transparent. It could also provide additional data and allow for a more thorough examination of the impacts along the supply chain.

The aim of this study is to frame P reporting in terms of sustainable global management of an essential global resource. Our objective is to review the current shortcomings of reporting along the global P supply chain, from exploration and mining of PR, to fork and waste. The research questions we ask are as follows: (1) “What are the issues and potential solutions with reporting along the global P supply chain, as derived from the literature?” and (2) “How is the P reporting process connected to reporting on the implementation of global sustainability initiatives such as the UN Sustainable Development Goals (SDGs)?”

## Materials and methods

We analyse the literature in detail and identify the shortcomings of the current supply chain reporting. We started from the search string and databases shown in Table 1 and we subsequently used snowballing to access other studies that were not immediately visible. Overall, in our results we used 18 academic journals and books, nine documents from reporting entities, five reports from Non-Governmental Organisations and one legal document.

**Table 1:** Researched databases and search strings used for literature review and snowballing

Researched databases	Search string in databases
• Stockholm University Library e-resources	• Phosphorus/phosphate resources
• Scopus	• Phosphorus/phosphate reserves
• Science Direct	• Phosphorus/phosphate deposits
• SciFinder	• Phosphorus/phosphate/fertilizer reporting
• SpringerLink	• Phosphorus challenge
• National and University of Iceland Library e-resources	• Peak phosphorus
• Google Scholar	• Phosphorus/phosphate/fertilizer losses
• Food and Agriculture Organisation Statistics	• Phosphorus/phosphate supply chain
• World Bank Open Data	• Phosphorus/phosphate/fertilizer externalities
• United States Geological Survey—Commodities	• Phosphorus/phosphate/fertilizer pollution
	• Phosphorus/phosphate + Western Sahara
	• Phosphorus/phosphate + conflict
	• Phosphorus/phosphate/fertilizer data
	• Phosphorus/phosphate governance
	• Eutrophication + phosphate/phosphorus
	• Eutrophication + fertilizers
	• Eutrophication + global, food waste/wastage
	• Food waste/wastage + global

For P resources and reserves, the study addresses exclusively P derived from phosphate rock. For the other parts of the supply chain, we refer to all P input, including non-PR sources. The study starts from the idea that public knowledge on all aspects of the P supply chain should be basic knowledge for a basic right: access to food (Wellmer and Scholz 2015). The human right to adequate food is embedded in the UN International Covenant on Economic, Social and Cultural rights and is defined as follows:

The right to adequate food is realized when every man, woman and child, alone or in community with others, has the physical and economic access at all times to adequate food or means for its procurement (FAO 2012).

Our conceptualization of the P supply chain relies on the use of systems analysis and systems thinking to frame our results. Systems thinking is an approach that “embraces the nature and organization of coupled human and natural components, actors and relationships” (Bunch et al. 2018). In particular, we use one of systems thinking conceptual tools, a flowchart (Morecroft 1982), to better illustrate the linear yet complex aspects of the global P supply chain and differentiate between its main sectors. This tool is needed due to the cross-sectoral and multi-actor characteristics of the global P supply chain. In Discussion section, we use systems thinking to build our arguments about the implications and relevance of reporting on the global P supply chain in terms of global sustainability and global food security.

## Results

We identified four major issues in P reporting in the literature synthesis: (1) reporting P as a resource; (2) reporting inefficiencies and losses along the global P supply chain; (3) the extent of global P supply externalities and their monitoring; and (4) the implications of a lack of open access to data.

### Deposits, resources and reserves

A number of studies advanced the concept of peak phosphorus following the 2008 price crisis, most notably Cordell et al. (2009) and Ragnarsdottir et al. (2011). In 2010, the USGS reported a nine-fold increase in Moroccan reserves, from 5.7 to 51 billion tonnes (USGS 2011). The change occurred due to USGS adopting a 2010 reporting methodology proposed by the International Fertilizer Development Centre (IFDC) with the scope of ending the “peak phosphorus” debate (IFDC 2010). As expected, the event prompted critics to indicate that peak phosphorus had been based on static, unchanging estimates by USGS on PR reserves and reserves base (see Table 2), a method not suitable to assess the longevity of PR world deposits (Scholz and Wellmer 2013). However, Edixhoven et al. (2014) criticized the new IFDC methodology, highlighting that the new reporting methodology renders the PR reporting inaccurate, by allowing “deposits to be termed reserves or resources which could not be recognized as such under leading mineral resource classifications” (Edixhoven et al. 2014). The post-2010 definition of “reserve” according to USGS was also modified by removing the legal aspect of an ongoing or potential resource extraction (see Table 2). In sect. “Socio-political externalities”, we show how this can have implications for some PR deposits, such as the ones in the contested Western Sahara region. Of note is that publicly available data are scarce when it comes to global PR reserves and resources. At present, USGS is the only agency publicly reporting on a yearly basis on global PR reserves and resources.

**Table 2:** Most common reporting terminology for P as a resource

Preporting terminology	Definition
Phosphate rock (PR); also phosphorite	Rock with a high concentration of phosphates in nodular or compact masses. Here, phosphates can include any of the 200 recognized species of phosphate minerals (Britannica 2006)
Phosphate rock mineral deposits	A mineral occurrence of PR, sufficient size and grade that it might, under the most favourable of circumstances, be considered to have economic potential (USGS 1996)
Ultimately recoverable resource	The amount of resource that is eventually extractable—including high grade, medium grade, low grade (Sverdrup and Ragnarsdottir 2014); Resources that can be extracted with future technologies, with either lower extraction costs or an increase in the potentially producible quantities (Speirs et al. 2015)
Phosphate rock ore deposit	A mineral deposit of PR that has been tested and is known to be of sufficient size, grade and accessibility to be producible to yield a profit (USGS 1996)
Phosphate rock reserves, ante-2010 USGS definition	That portion of an identified resource from which a usable mineral or energy commodity can be economically and legally extracted at the time of determination (USGS 1980)
Phosphate rock reserves, post-2010 USGS definition	That part of the reserve base, which could be economically extracted or produced at the time of determination. The term reserves need not signify that extraction facilities are in place and operative. Reserves include only recoverable materials; thus, terms such as “extractable reserves” and “recoverable reserves” are redundant and are not a part of this classification system (USGS 2010)
Phosphate rock ore grades (in P_2_O_5_ as % of PR)	The concentration of PR within the ore. Grade may exhibit considerable variation throughout the deposit (Britannica 2007)
Cut-off grade	Grade below which is not profitable to mine PR even though P_2_O_5_ is present in the ore (Britannica 2007)
Phosphate rock Concentrate	Crushed PR after beneficiation (increased P_2_O_5_ concentrations)

Table 2 summarizes the significant differences between currently used terminologies. At present, PR reporting—including for the USGS—relies on country- or deposit-specific assessments. In undertaking these assessments, geological surveys or companies can use different terminologies and, in some cases, different methodologies. This assessment approach decreases the reliability of global PR reporting. A relevant example is the 2005 compilation of studies by the International Geological Correlation Programme (IGCP). In this book, all currently identified phosphate deposits of the world are described, country by country. Each deposit is further divided into assessments of ore bodies. However, the methodologies and terminologies used to calculate PR reserves and resources vary from country to country and sometimes among ore bodies of the same deposits.

Moreover, the characteristics of one deposit or ore body can be very vague, for instance, the 800 million tonnes Saudi deposit at Al Amud, which has an ore grade of “less than 20% P_2_O_5_” (Notholt et al. 2005), or the ore body at Constable Hill in the Western Cape Province of South Africa, which has 0.27 million tonnes at 27.5% P_2_O_5_ concentration, “with an additional several million tonnes of low-grade ore” (Notholt et al. 2005).

Access to accurate, up-to-date data is also restricted, not only to the public but also to reporting entities. This is in part due to the concept of proprietary data. In Australia, for instance, IFDC (2010) noted that the state geological survey does not have a complete account of the country’s PR reserves and production because mining and fertilizer companies are not obliged to provide this information. Disclosure of PR reserves, resources and production can be problematic when a state considers this information of national security. China, for instance, has in the past altered its reported reserves without explanation. Its reserves doubled over night when it joined the World Trade Organisation (WTO) in 2001 and decreased in 2007–2008, when the fertilizer spike in prices occurred (Cordell and White 2011). Therefore, reporting entities often need to estimate a country’s resource.

Rosemarin and Ekane (2016) have identified this lack of transparency and best practices in terms of PR reporting and highlighted the need for more global governance and more involvement from the UN when it comes to reporting of P resources. Their perspective is that UN oversight can work towards more cooperation and trust between reporting parties and improve the accuracy of global PR reporting. These authors propose a Global Phosphorus Facility (GPF), under the lead of UN Environment Programme (UNEP) to “provide clarity on the geological knowledge base as well as on best practices along the entire value chain” (Rosemarin and Ekane 2016). Similarly, they argue that other supra-national institutions can increase the transparency and reliability of global PR reporting by establishing their own reporting mechanisms. Cordell and White (2011) also argue that reliance on USGS reporting does not allow for triangulation of results with other sources and there is a need for other entities to conduct their own reporting to allow for comparison.

Table 3 summarizes the PR reporting issues, its effects and possible solutions, based on existing literature. In general, more leadership by the UN as the ultimate global partnership platform can lead to a more transparent and accountable reporting. The International Resource Panel of UNEP for instance uses reporting data from USGS when discussing P (UNEP 2019). Other global reporting entities are also essential in strengthening global governance of P by following a unified and responsible, transparent and reliable reporting process (Rosemarin and Ekane 2016). National geological surveys, mining and fertilizer companies, the IFDC or IFAD should aim to harmonize their terminologies and definitions when reporting. In this way, global resources can more accurately be estimated and researchers, policy makers and the private sector can make more informed decisions and carry out research with a lower degree of uncertainty.

**Table 3:** PR reporting issues, their effects and proposed solutions

Preporting issue	Effects	Solution	Actors
Different definitions of reserves and resources	Non-reliability of reserve/resource estimates and incompatibility between reports of different reporting entities	Harmonization of terminology	Reporting entities
No differentiation between mineral ore and phosphate concentrate	Can lead to overestimation of reserves/resources	Specification of which of the two is being reported	Reporting entities
Lack of reporting due to proprietary data or national security concerns	Non-reliability of reserve/resource estimates	Global governance in reporting for a more transparent reporting	UN, other supra-national institution, global reporting entities
Dependence on one publicly available, open access source of annual reporting	Lack of transparency and accuracy of what is being reported and who reports to the reporting entity, non-reliability of data	Open access annual reporting from a number of reporting entities to allow triangulation of results	UN, other supra-national institution, global reporting entities

### Losses and inefficiencies along the supply chain

Reporting along the P supply chain could also help in increasing P use efficiency, while at the same time considerably reducing P losses. Scholz and Wellmer (2015) estimate that only 10% of the phosphorus used for agro-food production is digested by humans. Cordell et al. (2009) also suggested that as much as 80% of the P is wasted from mine to fork, but due to lack of reliable and accurate data, it is difficult to quantify losses at each step of the value chain.

**Fig. 1:**
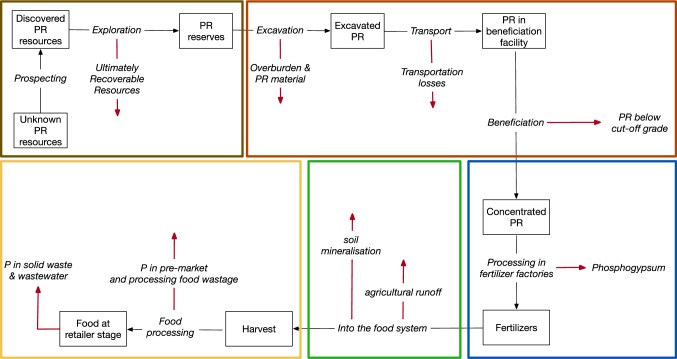
Flowchart of the global P supply chain with P losses along the chain. Red arrows represent losses, and coloured squares represent different sectors of the P chain

Figure 1 shows a flowchart of the P supply chain, from resource exploration to waste, with its associated losses along each supply chain step, as derived from the literature. In the dark brown box, the prospecting and exploration processes at the initial stage of the value chain are subject to the limitations in deposit characterization and reporting. This poses challenges in determining the amount of ultimately recoverable resources (URR). Steiner et al. (2015) proposed solutions to increase the efficiency of exploration. These include improved geophysical methods, re-exploration of P in search of other resources such as uranium, and search strategy optimization. Actors involved at this stage would be geological surveys and mining companies.

The next supply chain sector (Fig. 1) is mining and beneficiation, in light brown. Data on how much P is lost in the overburden, during transport or during beneficiation, can be provided at the mine and beneficiation unit level. Actors involved here are the mining companies and the authorities responsible with the regulation of mining activities.

Next, the blue box (Fig. 1) covers the processing of beneficiated concentrate to fertilizer. Fertilizer production is highly inefficient, as “between 30 and 50% of the P_2_O_5_ equivalents in the mined ore is unrecovered and is contained in waste ponds” (IFDC 2012). However, improving estimations would require an integrated reporting from the fertilizer producing companies. Proprietary data and lack of monitoring and reporting regulations make this difficult.

In the green box (Fig. 1), phosphate fertilizers are spread on agricultural land and follow three paths: (1) absorption by crops, (2) accumulation in soil through mineralization, and (3) runoff or transport by subsurface drainage in water bodies (King et al. 2014). The amount of P in absorbed crops can be estimated by the harvested crop amounts. However, while some studies investigated mineralization of organic P in soil at a global level (Bunemann 2015), studies examining the extent and characteristics of inorganic P mineralization are limited to some soil types or some geographical regions (see Achat et al. 2016). Similarly, literature on the amount of P runoff and subsurface drainage is also limited to region- or soil-specific studies (see King et al. 2014).

The yellow sector (Fig. 1) of the P chain is food production and consumption. Some recent studies investigate P losses specifically in this sector at a country level (e.g. Wang et al. 2018 for China). More studies investigated and reviewed the extent of post-harvest food wastage at the farm, manufacturer, retailer and transportation stages of the food supply chain (e.g. BCG 2018). The information could be used to calculate and quantify the extent of P losses. At the very end of the yellow block is the waste from food shops/supermarkets and consumers, which comes in the form of both food waste and wastewater. In some parts of the world, stricter water protection regulations have led to an increasing awareness of the double role of P as both a resource and a pollutant. In Europe, for instance, end of pipeline studies showed P from wastewater could supply up to 20% of the European demand (European Commission 2017). The earlier stages of the supply chain, however, remain largely unreported and thus their recovery potential remains untapped.

Table 4 summarizes the reporting issues for each of the P supply chain sectors, proposed solutions and actors responsible for their implementation, as derived from the literature. In general, reporting on P management can be implemented and monitored by a range of actors, from both the public and private sector. This includes companies involved in mining PR deposits, the food industry, fertilizer companies, state departments, environment agencies, municipal authorities but also “consumers”, such as farmer associations or wastewater treatment plants. The wide range of actors involved in the supply chain highlights the need of working across silos for an integrated reporting process.

**Table 4:** Reporting issues, solutions and involved actors for the global P supply chain sectors

P supply chain sector	Reporting issue	Solutions	Actors
Prospecting and exploration	Lack of data, different geological exploration methodologies	Improvement and harmonization of exploration methodology, sharing data	Mining companies, geological surveys, state authorities/departments responsible for the mining industry
Mining/excavation and beneficiation	Lack of data, considered proprietary data or sensitive information at state level	Sharing data on mining and beneficiation	Mining companies, state authorities/departments responsible for the mining industry
Fertilizer processing	Lack of data, considered proprietary data or sensitive information at state level	Sharing data on fertilizer processing and the composition processing by-products	Fertilizer producing companies, national environment agencies
Agricultural application	No reporting on the extent of P runoff from agricultural land to water bodies or of P mineralization in soil	Implementation of a P runoff monitoring programme on agricultural lands and reporting on estimates of P mineralization in soil	Farmers, state departments/authorities responsible for agriculture, national environment agencies
Post-harvest	Lack of data—in many cases considered proprietary data—on the extent of post-harvest food wastage; no reporting on the extent of P in solid municipal waste	Sharing of post-harvest food wastage data; estimates of the P in solid municipal waste	Food processing companies, retailers, food agencies at state level, municipal authorities responsible for waste, national environment agencies

### Mine to waste externalities

#### Environmental externalities

A 2019 cradle-to-grave analysis of phosphorus fertilizers by UNEP’s International Resource Panel (IRP) revealed increasing negative impacts of PR mining, fertilizer production and application. Phosphoric acid production and PR mining are responsible for greenhouse gas emissions, largely through energy use. Ecotoxicity, human toxicity and eutrophication are caused by fertilizer application and, to a lesser extent, by PR mining. Finally, air pollution is mainly caused by phosphoric acid production and PR mining. In all cases, the negative impact of cradle-to-grave processes in phosphorus fertilizers have increased by 20% from 2000 to 2015 (IRP 2019). Reporting on the environmental impacts of phosphate mining is thus essential in protecting biodiversity, water and soil resources, and the climate. Ecosystems can be critically damaged by PR mining, with negative effects for the environment, society and economy.

P is also responsible for alarming rates of worldwide eutrophication. Freshwater basins covering 38% of the global land cover have P water pollution levels higher than those basins can assimilate (Mekonnen and Hoekstra 2017). Eutrophication leads to the creation of so-called “dead zones” by enabling overgrowth of algae, which block the inflow of oxygen into the water. Eutrophication can also negatively affect the use of water for human purposes, including provision of drinking water and economic activities such as fishing.

#### Socio-political externalities

Significant PR resources are found in the disputed region of Western Sahara, which in 2016 accounted for almost a quarter of all PR exports of Morocco (OCP 2017). Western Sahara has been in a conflict since 1975, when most of the region was occupied by Morocco, while the remaining part was claimed by the Polisario Front, which installed the Sahrawi Arab Democratic Republic or SADR (Saul 2015).

Morocco has repeatedly been accused of violating the human rights of the Sahrawi people, indigenous to Western Sahara, as well as violating international law by exploiting resources from an occupied territory (Cordell 2015; Saul 2015; Amnesty International 2018). Western Sahara Resource Watch (WSRW) reported that the number of Sahrawis employed at the Bou Craa complex decreased from 1600 jobs in 1968 to only 200 in 2011 and that Sahrawis are discriminated against Moroccans (WSRW 2011). On the other hand, the Polisario Front has been accused of failing to hold to account those responsible of violating human rights in its camps during the 1970s and 1980s (Amnesty International 2018). Two rulings of the European Court of Justice in 2016 and 2018 decided that the Association and Liberalisation Agreements in agriculture and fisheries concluded between the EU and Morocco did not apply to Western Sahara, as the region has a separate and distinct status guaranteed under the Charter of the United Nations. The Court highlighted that it was not apparent the people of the territory of Western Sahara consented to the EU-Morocco agreement, although they had the status of a third party (CURIA 2018). Some fertilizer companies also acted on the matter of phosphate originating from Western Sahara. For example, two of the three importing companies in Australia stopped purchasing PR originating from Western Sahara as of 2015, soon followed by fertilizer companies from Norway, Germany, the Netherlands, Belgium, Uruguay, Switzerland and the US (WSRW 2017).

Court rulings and reports from organizations such as WSRW and Amnesty International are examples of how reporting on the socio-political impact of PR mining can influence the behaviour of various actors in reducing these impacts. Monitoring programmes and periodical reports of the two NGOs informed governments, companies and investment funds about the Western Saharan origin of PR and allowed them to make informed ethical decisions. By ruling on the legality of PR exploitation, court decisions also influence the activities of those involved in the P supply chain. At the same time, court rulings can indicate areas in the supply chain where more reporting and monitoring is needed.

Most of the PR-rich countries score low to very low in the Corruption Perception Index. The index ranks countries from 1 to 176 with 1 being the least corrupt. China is ranked 77th, Morocco 81st, Algeria 112th, Egypt 117th and Russia is at 135th (Transparency International 2019). When it comes to the World Bank’s Worldwide Governance Indicators, all the above countries score low or very low on the control of corruption, rule of law, political stability/no violence and the voice and accountability indicators (World Bank 2019). These indexes and indicators can thus indirectly inform the underlying ethics of P-procurement decisions by government, companies, consumers or the general public.

### Open access to data

Open access data have been advocated in the literature as a tool to improve governance, including governance of natural resources (see Attard et al. 2015). Governments are usually seen as the entities that should provide open access to their data, to increase transparency but also to enable interested and affected stakeholders to reuse, redistribute and innovate on the data provided (Attard et al. 2015). Such transparency makes governments more accountable to their actions and enables citizens to actively participate in the governance process (Attard et al. 2015). However, companies can also provide access to their data. Carbonell (2016) has called for the use of big data by companies in big agriculture to evaluate and monitor externalities of the industrial agriculture system. The author argues that this would enable research on the designation of best agriculture models for the future of global food production.

With a similar scope, Cordell and Neset (2014) advanced the Phosphorus Vulnerability Assessment Framework, through which they identified and integrated 26 phosphorus-related biophysical, technical, geopolitical, socio-economic and institutional factors that can lead to food system vulnerability. In a later paper, Cordell and White (2015) addressed global phosphorus vulnerability indicators in the global food system and suggested a number of publicly existing databases to track progress on these indicators. Their sources are in general international reporting entities, such as the World Bank, the International Fertilizer Association (IFA), the Food and Agriculture Organisation (FAO), but also “domestic sources” (Cordell and White 2015).

Wellmer and Scholz (2015) brought into discussion the population’s “right to know”. In formulating their argument, the authors cite population’s right to know as a “basic regulatory rule in the frame of democratic and free market-based societies” (Wellmer and Scholz 2015). As such, public knowledge about phosphorus, which is an essential fertilizer, should be basic knowledge for the basic right of access to food.

However, open access to data does not in itself guarantee such benefits. Jansse et al. (2012) warned about the barriers of benefitting from open data, such as task complexity in processing the data. The authors stress that “open data has no value in itself; it only becomes valuable when it is used”. Transforming open access public data into some form of public value has been researched to an insufficient extent (Jansse et al. 2012).

Open access to P reporting can not only assist in tracking vulnerability and impact of the value chain, but also help in tracking progress on broader indicators, in which P plays a significant role. For instance, despite the fact that P supply chain effects and has a central value in food production, P reporting is not an integral part of the reporting for the UN Sustainable Development Goals (SDGs). In Table 5, we make the connection between reporting on achieving the SDGs as a global sustainability framework and reporting on the P supply chain.

**Table 5:** Connection between SDGs and reporting on the P supply chain

Sustainable Development Goal	How reporting on the P supply chain affects reporting on the fulfilment of the goal
SDG1—Zero poverty SDG2—Zero hunger SDG3—Good health and well-being	- Poverty, hunger and health are related; people in less developed countries spend from 30 to 56% of their budget on food (WEF 2016) - Rural population in less developed countries is highly dependent on the productivity of their subsistence and semi-subsistence agriculture, and therefore P input can be essential - Eutrophication through P pollution can negatively affect the use of water for human purposes, including provision of drinking water. It can also negatively impact fishing, leading to decreased food availability and decreasing economic revenues
SDG6—Ensure availability and sustainable management of water and sanitation for all	- P pollution as runoff or wastewater effluent/sewage and its associated eutrophication (see section “Environmental externalities”)
SDG12–Responsible Consumption and Production	- High rates of losses along the P supply chain (see section “Losses and inefficiencies along the supply chain”)
SDG14–Life under water	- Eutrophication and dead zones due to P pollution (see Sect. 3.3.1)
SDG16—Peace, justice and strong institutions	- Oligopolistic phosphate market moving towards a monopoly with phosphate rock from conflict regions (see sect. “Socio-political externalities”)

In general, reporting on the P supply chain allows a better reporting on food security, pollution and human well-being (Cordell and White 2015) and all of these sectors are at the core of most of the SDGs. In turn, this can enable a better and more informed policy-making process in these areas but also an increased awareness among the public and other affected actors, such as farmers.

## Discussion

In Results section, we showed that reporting on the global P supply chain has a number of critical flaws. This virtually makes the supply chain a black box, the contents of which are difficult to predict or analyse. The different terminologies used to report on P deposits means that current estimates on the actual P resources are inaccurate. Moreover, lack of global, public reporting entities with the exception of the USGS do not allow for triangulation of results. Sudden changes in methodologies can have serious repercussions on the global P market, influencing the price of phosphate rock and P fertilizers. It also poses serious challenges in the evaluation of global P scarcity and raises questions as to how critical a material P actually is. A clear example in this sense is the 2010 overnight change in the reserves of Morocco and Western Sahara by IFDC, which had previously been considered potential resources.

Reporting on losses along the P supply chain is one of the most important processes that can allow for an increase in efficiency in the way P is being mined, processed, applied and recovered. It is also one of the most challenging reporting processes because it would require a wide range of actors from across sectors, local, national and supra-national, to monitor and publish their data. Perhaps an even greater challenge is to harmonize and integrate all data once it has been collected and made available. The benefits, however, would also be substantial, considering that 80–90% of the mined P is lost before reaching human consumption through food.

Although P has an extensive supply and value chain, there have been very few attempts at investigating this chain’s externalities, both environmental and social. We discussed the externalities in sect. “Open access to data”, with a particular focus on Morocco. The North-African kingdom is emerging as a key player on the phosphate market and because of its vast reported phosphate reserves, its importance will only increase in the future. Access to reports on externalities in PR-exporting countries such as Morocco can allow not only governments, fertilizer companies, farmers but also investment companies and/or banks to make ethical, socio-environmental sound decisions when buying phosphate or investing in PR mining companies.

Last but not least, we discussed the open access aspect of global P reporting. At the moment, the scarce information on the P supply chain is not publicly available, being mostly treated as proprietary data by mining and processing companies. Public knowledge on the P supply chain is needed because of the essential role of P in food production, global food security and the human right to food. But P reporting is also needed for other global goals, such as the SDGs. We directly linked the global P supply chain to seven SDGs, showing the interlinkages between the different impacts of the P chain and food security, pollution, environmental status, management of water resources as well as peace and justice. Reporting on the SDGs could improve through reporting on P, a natural resource that is key for seven of the 17 SDGs. Open access data on P reporting and the form publicly available data take are also important. Available data should be relevant and easy to analyse, which would make it valuable from a governance perspective. Access to relevant, easy to use data is essential to academia and civil society in their endeavour to complement the work of governments and other governance entities.

## Conclusion

Phosphorous is an essential resource for food production, but its supply chain has far reaching impacts on environment, society and economy. This study showed that when it comes to reporting on P reserves and resources, the information is not harmonized, unreliable, fragmented and non-transparent. This intransparency poses a fundamental threat to food security worldwide, influencing the price of P fertilizers and the ability of those in the food production system to sustainably plan for the future. The global P supply chain induces a number of environmental and socio-political externalities, which are poorly documented. Improving reporting on these aspects can assist policy makers, farmers, fertilizer companies, investment banks and the public to make informed, ethical decisions on the procurement of phosphate rock or P fertilizers.

Global leadership in P reporting can lead to a more integrated and transparent approach to the P supply chain. Working towards quantification of P losses and inefficiencies in the supply chain can lead to more sustainable production and consumption. It can also raise awareness about the importance of improved agricultural practices. Exposing P losses can not only translate into more accountability by all stakeholders involved in the chain, but it can also better inform policy makers across a variety of sectors, from agriculture, to waste management, innovation, pollution control and human rights protection.
